# Strangulated Hernia*Read before the Richmond Academy of Medicine and Surgery, July 28, 1896.

**Published:** 1896-09

**Authors:** Virginius W. Harrison

**Affiliations:** Richmond, Va.; Adjunct Professor of Practice of Surgery, University College of Medicine, etc.


### STRANGULATED HERNIA.*

By VIRGINIUS W. HARRISON, A. M., M. D., RICHMOND, VA.

Adjunct Professor of Practice of Surgery, University College of Medicine, etc.

In the study of this subject, we are considering one which, in a recent article before the Æsculapian Society of London, Mr. Stephen Paget says "lays before you a terrible list of the worst and most distressing cases that fall to the lot of a hospital surgeon." He refers to these cases in which the bowel is gangrenous, perforated or broken at the time of the operation. Of such cases he had twelve; four were inguinal; four were umbilical, three were femoral, and one in the left abdominal wall. One patient died during the operation, five a few hours afterwards, two died on the second day, one on the third day, one (aged seventy-one) lived ten days, one (an infant) lived for five weeks, one recovered. Having a condition occurring almost daily, or one that we may have to contend with at any time, and one which may lead to such untoward results, its discussion should be of benefit to us all.

By strangulated hernia we mean that a portion of the bowel, after passing through some opening (natural or otherwise) in the abdominal wall, has become constricted to such an extent that the circulation is interfered with, and, if not relieved, gangrene and death will result.

* Read before the Richmond Academy of Medicine and Surgery, July 28, 1896.

Strangulation may take place during some violent exercise, a knuckle of the bowel passing through some natural opening, and become constricted, or it may take place in an old hernial sac, by the addition of a fresh loop of bowel. It is not necessary that violent exercise should have originated the trouble, for this condition may be produced by an attack of diarrhœa, or dysentery, causing an increase of peristaltic action, congestion, swelling, and finally constriction by the engorgement of the bowel. There are other causes of strangulated hernia, such as injuries to the part by outward influences, impaction with fecal matter, etc., all too well known to consider at this time. The seat of stricture is usually at one of the hernial rings, though it may be anywhere in the sac, where inflammatory action has taken place, and new tissue formed a constricting band.

The local changes are those you would naturally expect when the circulation has been impaired or cut off—the amount of change being dependent upon the length of time, and completeness of the constriction. The symptoms as recorded in the text-books are said to be always about the same, viz: faintness, collapse, complete constipation, vomiting, a tumor at the site of strangulation, severe pain at this point, an absence of impulse in coughing, and the fact that you can not put the bowel back into the abdominal cavity easily, if at all. The pulse is a better guide than the thermometer, as the latter may indicate a subnormal or normal temperature.

Do not expect to find these symptoms in every case, for almost every writer on this subject records cases in which the symptoms are very mild. Paget, in his paper already referred to, records one case of a patient who had walked into the hospital, suffering no pain,—the operation proved the bowel to be already perforated, and of another who had had strangulated femoral hernia from Tuesday until Friday without being aware of the fact. She was thought to have had typhoid fever at first, as she did not refer to the hernia. At a further examination, the hernia was discovered, and the operation disclosed the fact that the bowel was perforated. This same gentleman incidentally refers to a case in his practice where the rupture did not take place at the seat of strangulation, but in a loop at some distance, which was bound together by old adhesions. DeGarmo admonishes us not to depend too much on local symptoms.

How shall we treat these cases? One writer goes so far as to say "the prognosis of these cases depends entirely upon the man who first sees them."

Preventive treatment by a radical operation would diminish the rate of mortality, but unless the pre-existing hernia is a source of great discomfort to the patient, we unfortunately cannot obtain their consent to an operation, and even our advice to always wear their truss is not heeded, owing to the discomfort they find in wearing one. So, until we can educate the people to the danger of neglect in these cases, we must await their presentation in a more dreadful condition, and use our best means for their relief, which is taxis and herniotomy.

We can not lay down any particular plan for the treatment of strangulated hernia any more than we could for any other condition in which the morbid anatomy is variable. The condition of our patient in general and the strangulated gut in particular, should engage our serious consideration, for to use taxis upon a gut already gangrenous or perforated would only consume time and place the patient in a far more serious condition. If we use taxis upon a case where suppurative peritonitis had taken place, and should be successful in replacing the gut into the peritoneal cavity, you can see it can but lead to a fatal issue.

Never use taxis when you suspect gangrene or perforation of the bowel, when the patient has suffered taxis at the hands of another, or in an old irreducible hernia. The results in these cases are more favorable in an inverse proportion to the amount of taxis. In a recent case taxis may be tried for a few minutes, and if unsuccessful, delay is inadmissble. Taxis may be tried under an anesthetic, but should be done with care, or we are apt to use more force and do more injury than would be done by an operation.

If taxis is unsuccessful, explain the true condition of your patient to his friends and himself, and tell them the necessity of early operative interference, or you may have said of you (as was said of a writer on this subject), "We took him to the hospital; the doctor operated immediately, without waiting to see if he was strong enough to stand the operation."

We are often tempted to delay an operation to see if temporizing and palliative treatment will not relieve the patient. Relief often comes by such method, but the undertaker often performs the greater service. When we wait for the severe symptoms, as was done a few years ago, before an operation was indicated, the hour of safety has passed, and the patient often doomed.

Even if our palliative treatment was successful in reducing the hernia, we would not see the condition of the bowel, and being aware that strangulation and perforation may take place after reduction, we think the patient would have a better chance of recovery by performing herniotomy. The various methods of operating I will not discuss. Choose that one which you think will give the patient the best chance to recover from the strangulation; and if the condition of the patient will permit, that one which will radically cure the hernia. Sometimes in a recent case, after cutting the stricture and pulling down the bowel so as to examine the point of constriction, if you will envelop the loop in a towel which has been wrung out of hot water—or better, a hot saline solution—you will see the circulation restored, and the bowel can be replaced in the abdominal cavity with a feeling of safety. Even in very old people this method is sometimes successful, as was illustrated recently in a case of Dr. McGuire, the patient being in the eighty-sixth year of his age.

If we encounter a case in which the bowel is suffering from suppurative peritonitis, gangrene or perforation, it would not be sufficient to follow the above method, and just what to do is often not easily determined. When the gut is gangrenous or perforated, of course there is but one thing to do, and that is to resect that portion of the gut; but an intermediate point between slight constriction and gangrene requires nice judgment.

The following conclusions by Dr. W. B. DeGarmo, in a paper on strangulated hernia, in the May number of *The Post-Graduate*, are of interest and worthy of study.

(1) Prompt operation saves complications and saves life.

(2) Infants seldom require operation.

(3) Medicine and external applications are dangerous, as their use causes delay.

(4) Operations done early are neither difficult nor dangerous.

(5) Rough handling is more dangerous than an operation.

(6) Morphia stops symptoms, but does not stop destructive changes.

(7) Local symptoms are misleading.

(8) Hot water saves resection, or furnishes prompt evidence of its necessity.

(9) Operate rather than attempt the reduction of a hernia acutely strangulated for twenty-four hours.

(10) Open to internal ring in every instance.

(11) Always draw the bowel down far enough to examine the point of constriction.

(12) It is not considered good practice to give cathartics after strangulation and return of the suspicious bowel.

In concluding my remarks, I wish to report a case operated on by me, terminating fatally. The history of the case, and results shown by post-mortem, were very instructive, and impressed upon me the importance of not returning to the abdominal cavity a gut which has not been examined, or a loop in which the circulation has not been fully restored.

On June 9, 1896, I was called at 6 P. M., to see W. G. W., *æt.* 55, white, a half-witted, but physically robust individual, with a good family history, as given by his brother. This man had done a great deal of heavy work, such as cutting wood and lifting.

I found him at my first and only visit to his home, suffering with a strangulated inguinal hernia of the right side. He had had for many years an irreducible hernia, but it had never given him any pain until thirty-six hours before I saw him. His brother had tried to reduce the rupture; being unsuccessful, he asked me to see him. After an examination of a very few minutes, I advised immediate operation, and sent him to the Virginia Hospital, at which place the operation was performed at 9:30 P. M.

The gut was found to be strangulated, and several places looked suspicious of beginning suppurative peritonitis. After cutting the stricture, and dissecting the adhesions of the sac, we enveloped the bowel in a towel wrung out of hot water, and continued to pour hot water over towel and gut for some time. The result was an improvement in the condition of the gut. After consultation, in which we were equally divided for and against resection, we decided to resect the bowel. While active preparations for this part of the operation were going on, our patient became profoundly shocked, and looked like he would not last long enough for us to close the wound, and as there was some doubt as to resection being imperative, I put the bowel back in the abdominal cavity and closed the wound, after allowing for free drainage by means of iodoform gauze.

June 10th, 11 A. M.—Patient reacted well from the operation. Temperature 99°; pulse, 80, and feeling very well. By 3 P. M., temperature was $102\frac{3}{5}$° F., pulse 100. One grain doses of calomel ordered to be taken until he had taken six grains. As this had no effect upon the bowels, I ordered a dose of Epsom salts, but this was not retained. At midnight, I ordered the calomel to be taken as before.

June 11th, 8 A. M.—No action from the bowels. An enema of glycerine was ordered, and was followed by a large action, and several later in the day. 10 P. M.—Temperature, 100°, pulse 90. The belly was swollen some in the morning, but was now reduced somewhat by the free movement of the bowels.

June 12th, 11 A. M.—Temperature 99°, pulse, 80; belly still swollen. 11 P. M.—Temperature $97\frac{4}{5}$°, pulse 80; belly not swollen at all.

June 13th, 11 A. M.—Temperature 99°, pulse, 80; symptoms of septic infection indicated by delirium. The external wound was found to be infected; the stitches were removed, and the wound washed with hydrogen dioxide (Oakland). 11 P. M.—The same condition of affairs, except the infection *seemed* less; the external wound looked better, which was again washed with the hydrogen dioxide.

June 14th, 11 A. M.—Temperature 98°, pulse, variable; sometimes full and regular; at other times feeble and irregular. His mind cleared and could recognize every one, but would not take his nourishment. This condition of affairs continued until 6 P. M., June 15th, when he died.

A post-mortem was held at 6:20, and revealed a coil of bowel adherent to the abdominal wound; the portion of bowel which was strangulated was found imbedded in a mass of pus and inflammatory exudate; the gut, though not perforated, was gangrenous, and broke down in the handling to remove it; the rest of the intestines were all matted by suppurative peritonitis.

I report this case to emphasize—

1st. The fact that we can not rely upon local symptoms to indicate the amount of damage that has been done by strangulation; nor will they indicate what is going on in the belly after an operation.

2d. That we can have septic peritonitis with a very small amount of fever, or even a subnormal temperature, a fairly good pulse, a very little pain, and the bowels acting freely; for in this case, though the post-mortem demonstrated a condition we would think to be sufficient to cause paresis of the whole intestinal tract, yet, after the first thirty-six hours, his bowels moved freely every day.

3d. When a bowel looks badly and there is doubt about the circulation being fully restored, the patient will have a better chance of recovery, by resecting the diseased portion of bowel.

I am confident had I continued the operation in the case reported above, the patient would have died before I had finished; yet I realized the fact, in my opinion, when I replaced the bowel in the abdominal cavity that his chance for recovery was very slight.

